# Shieldin complex assembly kinetics and DNA binding by SHLD3

**DOI:** 10.1038/s42003-023-04757-7

**Published:** 2023-04-08

**Authors:** Vivek Susvirkar, Alex C. Faesen

**Affiliations:** Biochemistry of Signal Dynamics, Max-Planck Institute for Multidisciplinary Sciences, Göttingen, Germany

**Keywords:** Biochemistry, Molecular biology

## Abstract

The Shieldin complex represses end resection at DNA double-strand breaks (DSBs) and thereby serves as a pro-non homologous end joining (NHEJ) factor. The molecular details of the assembly of Shieldin and its recruitment to DSBs are unclear. Shieldin contains two REV7 molecules, which have the rare ability to slowly switch between multiple distinct native states and thereby could dynamically control the assembly of Shieldin. Here, we report the identification of a promiscuous DNA binding domain in SHLD3. At the N-terminus, SHLD3 interacts with a dimer of REV7 molecules. We show that the interaction between SHLD3 and the first REV7 is remarkably slow, while in contrast the interaction between SHLD3 and SHLD2 with a second REV7 molecule is fast and does not require structural remodeling. Overall, these results provide insights into the rate-limiting step of the molecular assembly of the Shieldin complex and its recruitment at DNA DSBs.

## Abstract (web summary)

Within the DSB repression and resection complex Shieldin, SHLD3 binds a dimer of REV7, where the interaction with the first REV7 is the rate-limiting step in Shieldin assembly and DNA-DSB recruitment.

## Introduction

DNA double-strand breaks (DSBs) are highly toxic to cells as they cause full rupture of the chromosomes. Incorrect or a lack of repair leads to genomic anomalies ranging from insertion, deletions, duplications and translocations. These anomalies have been linked to embryonic death, early aging, genetic disorders, immunodeficiency, neurological disorders and cancer. During the G1 phase of cell cycle, the Shieldin complex is recruited in a 53BP1-RIF1-dependent manner and binds single-strand DNA (ssDNA) to shield the DNA from resection^1–4^. This commits the repair of DNA to the non-homologous end joining (NHEJ) pathway and thereby guards genomic integrity in an event where homology repair (HR) would be detrimental. The Shieldin complex has been identified as a key factor responsible for PARPi resistance in tumor cells lacking 53BP1. Therefore, understanding the molecular mechanism of Shieldin complex-mediated repair by NHEJ of DSBs is essential to understand the regulation of DNA repair in health and disease.

The Shieldin complex consists of four subunits, SHLD1 (205 residues), SHLD2 (904 residues), SHLD3 (250 residues), and HORMA domain REV7 (211 residues, also known as MAD2L2). The SHLD3-REV7 module is considered to be the recruitment arm, while SHLD2-SHLD1 is the effector arm of the complex^5^. SHLD2 is proposed to function as a scaffold protein and contains three putative oligonucleotide-binding (OB) folds that bind ssDNA^1,3^. The functions of SHLD1 and SHLD3 are unclear as they are biochemically uncharacterized. It has been proposed that Shieldin is not a constitutive complex, but rather that it is assembled and disassembled on-demand in cells^6–8^. Assembly of Shieldin might follow a linear hierarchy where SHLD3 localizes first to DNA breaks, followed by REV7 and SHLD2-SHLD1 in a 53BP1 and RIF1-dependent manner^2^. The molecular details of how Shieldin complex assembly is regulated and how it recognizes the complex variety of DNA substrates at site of DSBs remain unclear.

Regulating Shieldin assembly and disassembly is likely achieved through modulating REV7 metamorphosis. REV7 is a member of the HORMA (HOP1, REV7, MAD2) domain family, which are conserved signaling proteins serving as sensors in a variety of pathways, ranging from bacterial immunity, eukaryotic cell cycle, genome stability, sexual reproduction, and cellular homeostasis^9^. This family of metamorphic proteins has the rare ability to convert between topologically distinct folds (‘conformers’; as opposed to the common conformational changes) under physiological conditions^9^. Changing between conformers affects their ability to engage in protein-protein interactions, which would make the conversion between conformers obligatory for effector complex assembly and disassembly. Conformer switching is spontaneous but typically slow, due to the substantial unfolding and repositioning of structurally mobile elements at the N- and C-terminus to the static core fold of the HORMA domain^6^. The emerging paradigm for HORMA domain proteins is that they default to an inactive ‘open’ or ‘unbuckled’ state, before converting to a partner-bound active ‘closed’ state. This phenomenon thereby creates a rate-limiting step in effector complex assembly, which can be regulated or catalyzed by protein factors, like AAA^+^-ATPase Pch2/TRIP13. This controlled metamorphosis, which often involves a dimerization of metamorphic proteins, can therefore be used to control assembly and disassembly of effector complexes in space and time^9–11^.

The incorporation of a dimer of REV7 molecules in both Polζ and Shieldin is essential for their function in vitro and in cells^7,12,13^. Central to the interaction mechanism of ‘closed’ REV7 is the extraordinary ability to wrap its C-terminal tail (‘seat-belt’) around an interacting peptide motif of a client protein, which subsequently allows effector complex assembly. Using the ‘seat-belt’ mechanism, REV7 captures the REV7-binding-motifs (RBM) in SHLD3 (residues 49 to 62) and REV3 (RBM1, residues 1875 to 1896, and RBM2, residues 1991 to 2012)^12–14^. On the other hand, REV7 also interacts with the REV7 interacting motif (RIM) present on SHLD2 without directly involving the seat-belt. In this unusual state, where part of the seat-belt is ‘unbuckled’ and the C-terminal beta-strand of the seat-belt mimics the ‘closed’ state, the interaction between SHLD3 and a second REV7 molecule is mediated by SHLD2^2,15^. Despite structural differences and the retained ability to (albeit indirectly) interact with client proteins, this ‘unbuckled’ closed conformer was termed ‘open’, after the auto-inhibited ‘open’ default conformer state in MAD2^16^. It is currently unclear how many distinct conformers or stable intermediates REV7 can adopt, how the different conformers affect complex assembly, and how conformer conversion affects the kinetics of complex formation.

Here, we use a reconstituted recombinant human Shieldin complex consisting of full-length SHLD3, REV7 and the N-terminal peptide (1-95) of SHLD2 to study its binding to DNA and the consequence of REV7 metamorphosis on the kinetics of complex assembly. We show that this complex, which lacks the SHLD2 DNA binding domain, is able to bind to a variety of DNA substrates non-discriminatorily. We identify a DNA binding domain in the C-terminal part of SHLD3, and introduced point mutants of conserved residues in a predicted electropositive pocket in order to prevent DNA binding. Quantification of the interaction kinetics of REV7 with the RBM’s on SHLD3 and REV3 using Surface Plasmon Resonance (SPR) and a fluorescence polarization sensor, shows a conserved and slow capture by the seat-belt of the HORMA domain. In contrast, the interaction of the second REV7 molecule to REV7-SHLD3-SHLD2 is fast in a conformer-independent manner. These observations support a model where the DNA binding domain contributes to the initial recruitment of SHLD3 and that the assembly of the Shieldin complex is rate-limited by the incorporation of only the first REV7 molecule.

## Results

### SHLD3 has a DNA binding domain

**Fig. 1:**
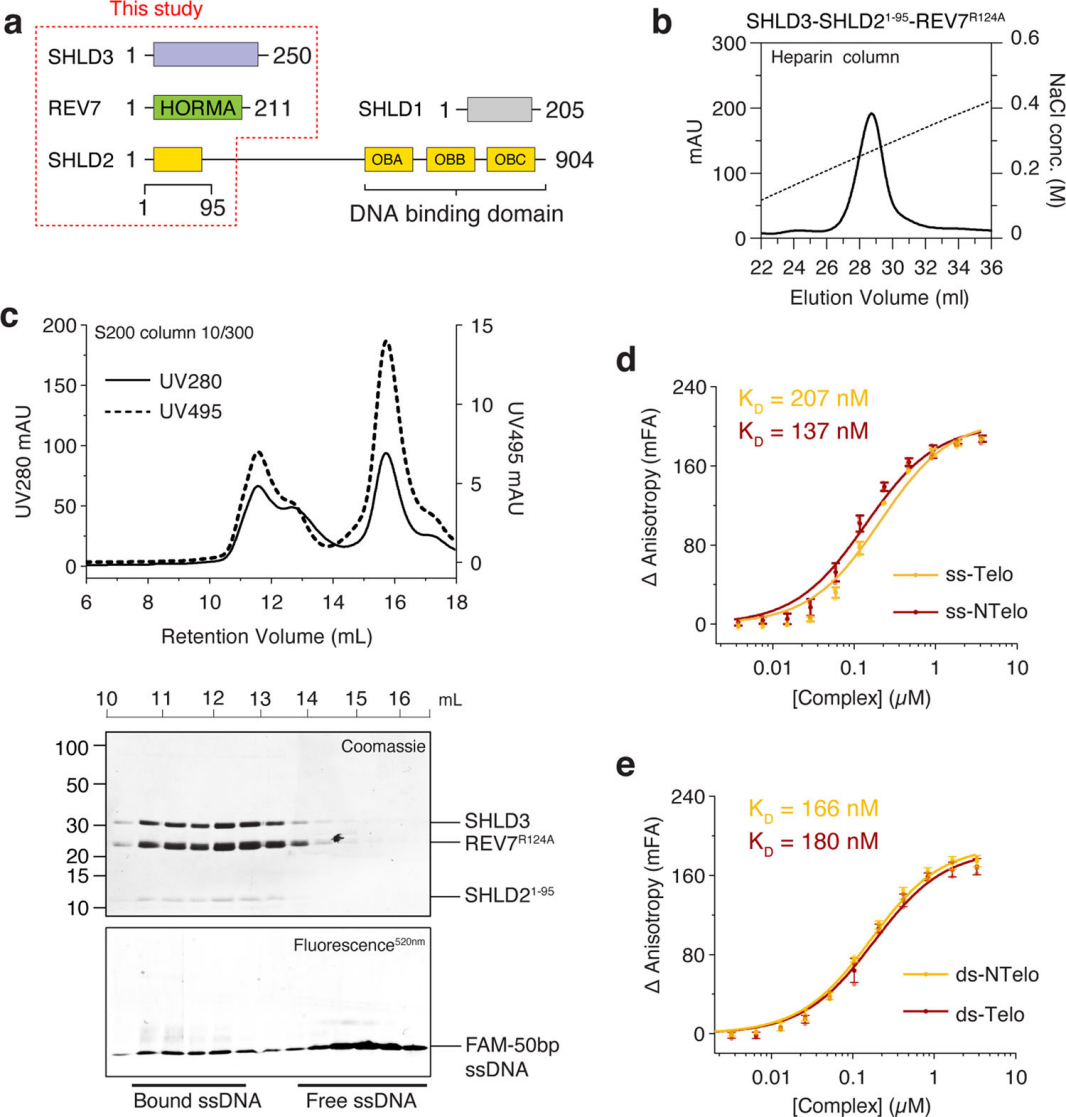
SHLD3-SHLD2^1–95^-REV7 ternary complex binds DNA. **a** Schematic drawing of the Shieldin complex containing SHLD1, SHLD2, SHLD3 and REV7. Protein constructs present in this study are highlighted. **b** Heparin column elution profile of the SHLD3-SHLD2^1–95^-REV7 complex. Point mutant R124A generates a dimerization-defective REV7 variant. **c** The SHLD3-SHLD2^1–95^-REV7 complex co-migration with ssDNA (Supplementary Tables 1, 2) in size-exclusion chromatography. **d**, **e** Fluorescence anisotropy titration experiments of Shieldin complex with (**d**) ssDNA (non-telomeric and telomeric) and (**e**) dsDNA (non-telomeric and telomeric). Error bars represent s.d. (*n* = 3 independent experiments). See Supplementary Tables 1, 2 for DNA details.

To understand the molecular assembly mechanism of Shieldin and its recruitment to DSBs, we purified a stable ternary complex (called Shieldin hereafter) consisting of human full-length SHLD3, a N-terminal peptide containing the first 95 residues of SHLD2 (SHLD2^1–95^) and REV7 (Fig. 1a). Although this complex lacks the DNA binding OB-fold domains in SHLD2, we noticed that the complex could bind to a heparin column, which suggests an ability to bind DNA (Fig. 1b). To test for DNA binding, we incubated the Shieldin complex with an excess of 50 bp 5(6)-carboxyfluorescein (5(6)-FAM) labeled ssDNA and carried out analytical size-exclusion chromatography (SEC). In the presence of the Shieldin complex, the elution peak of labeled ssDNA moved to a higher apparent molecular weight and co-migrated with Shieldin, suggesting the formation of a Shieldin-DNA complex (Fig. 1c). Next, we used the FAM-labeled DNA to carry out fluorescence anisotropy (FA) measurements to quantify the binding affinity of the Shieldin complex for different DNA substrates. No major differences in dissociation constant (K_D_) were observed between ssDNA telomeric (207 nM), ssDNA non-telomeric (137 nM), dsDNA telomeric (180 nM) and dsDNA non-telomeric (166 nM), nor with DNA substrates with 5′ or ′3 overhangs (varying from 15 to 1 bp overhang), of which some included DNA hairpins (144 nM to 576 nM) (Figs. 1d, e, S1a–c).

**Fig. 2:**
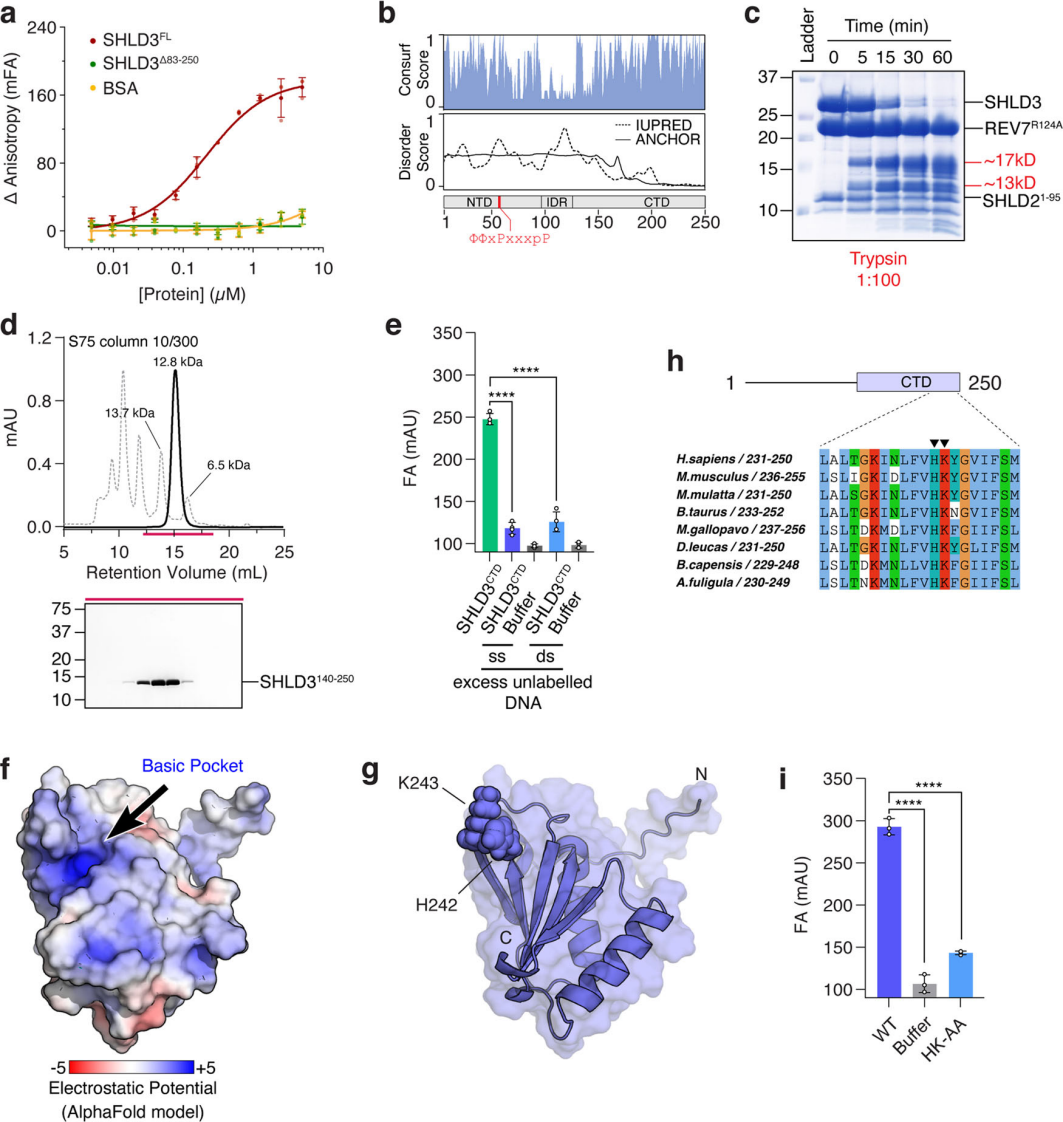
SHLD3 C-terminus contains a DNA binding domain. **a** Fluorescence anisotropy measurement of different constructs of Shieldin complex for binding affinities against ssDNA (Supplementary Tables 1, 2). Shieldin complex with deletion of SHLD3 residues 83–250 is deficient in DNA binding. Brown line represents Shieldin complex, green line represents Shieldin complex^ΔSHLD3(83-250)^, yellow line represents BSA. Error bars represent s.d. (*n* = 3 independent experiments). **b** Disorder prediction of SHLD3 using IUPRED shows that the N-terminal region (1–150) is disordered while the C-terminal region is folded. The sequence conservation score shows increased conservation in the N-terminal region (1–83) and C-terminal region (140–250). **c** Proteolytic digestion of the Shieldin complex with trypsin shows degradation of SHLD3 to stable fragments of roughly 17 kDa and 13 kDa. **d** Purification of SHLD3^140–250^ by size-exclusion chromatography. **e** FA competition assay shows SHLD3^CTD^ binds ssDNA and dsDNA without preference (Supplementary Tables 1, 2). FAM-labeled ssDNA at 10 nM was incubated with 1 µM SHLD3^CTD^. Labeled ssDNA was competed out using either 1 µM non-telomeric ssDNA or dsDNA. Error bars represent s.d. (*n* = 4 independent experiments). Two-tailed Student’s test are indicated: ****p < 0.0001. **f** Surface electrostatic analysis of the SHLD3^CTD^ AlphaFold2 model reveals presence of an electropositive patch. **g** SHLD3^CTD^ shown in surface representation with H242 and K243 shown as spheres. **h** Residues H242 and K243 are conserved across SHLD3 homologues in higher eukaryotes. Sequence alignment was performed with Clustal package in Jalview^43^. Residues are colored according to Clustalx scheme, where conserved residues are colored as follows: blue (hydrophobic), red (positively charged), orange (glycine), cyan (hydrophobic) and green (polar). **i** Introduction of H242A and K243A point mutants abolishes binding affinity of SHLD3^CTD^ to ssDNA (Supplementary Tables 1, 2). Experiment performed as in (**e**).

To determine which component in the Shieldin complex contains this promiscuous DNA binding domain, we tested different Shieldin subcomplexes for DNA binding. After incubation with complexes lacking SHLD3, we observed a loss of FA signal which suggested that the ability to bind to DNA was lost (Fig. S1d). Since the SHLD3 N-terminus is involved in REV7 binding, we suspected that the DNA binding domain was in the C-terminal part of the REV7. This region is predicted to contain a translational elongation initiation factor EIF4E-like domain^4^. To test this, we generated a truncation mutant that lacks this SHLD3 region (84–250) (Fig. S1e). As expected, this Shieldin complex failed to bind ssDNA (Fig. 2a). To understand the molecular mechanism of DNA recognition by SHLD3, we sought to identify and purify the DNA binding domain. Analysis of sequence conservation reveals high levels of conservation in the C-terminal region of SHLD3^17^. This coincided with predicted secondary structure in this region, suggesting a conserved and folded domain (Fig. 2b)^18^. We carried out limited proteolysis with trypsin and observed two stable fragments of SHLD3 corresponding to ~17 kDa and ~13 kDa (Fig. 2c). We designed a 13 kDa SHLD3^CTD^ construct (SHLD3^140–250^) based on the secondary structure prediction and sequence conservation, which we subsequently expressed in *E.coli* and purified to homogeneity (Fig. 2d). We tested it for DNA binding using a FA competition assay, where we pre-incubate SHLD3^CTD^ with labeled ssDNA and subsequently monitor the loss of FA signal in the presence of an excess of unlabeled competing ssDNA or dsDNA. This showed that SHLD3^CTD^ can indeed bind DNA with no discernible preference for ss- or dsDNA (Fig. 2e). This was confirmed in titration experiments, where the measured binding constants of SHLD3^CTD^ to the tested DNA constructs were similar to the Shieldin complex (Fig. S1f, g). Next, we used Alphafold2 to model the structure of SHLD3^CTD^^19^. SHLD3^CTD^ was modeled using ColabFold with default settings, which generated a high-confidence 3D model (pLDDT score of greater than 90) (Fig. S1h)^20^. As expected, the predicted model of SHLD3^CTD^ showed a high degree of homology to translation initiation factor EIF4-E (Fig. S1i). Surface analysis showed the presence of an electropositive patch that includes the conserved residues H242 and K243 (Fig. 2f–h). Mutating H242 and K243 to alanine showed a strong reduction of signal in the FA assay using ssDNA and both double- and single-stranded telomeric DNA, which suggests a general loss of DNA binding (Figs. 2i, S1j). Taken together, we identify SHLD3 as a promiscuous DNA binding protein within the Shieldin complex.

### REV7 dimer in Shieldin requires only a single defined conformer

The Shieldin complex stably integrates an asymmetric dimer of REV7 confomers^15^. Dimerization is a common feature of metamorphic proteins, including HORMA domains, which can aid in the transition between conformers. For example, the asymmetric dimerization of an ‘open’ and ‘closed’ conformer of HORMA domain protein MAD2 was shown to be essential in the mechanism to accelerate the conversion between conformer states and thereby catalyse the formation of the Mitotic Checkpoint Complex^10,21^. However, it is currently unclear if the different REV7 conformers can dimerize independently of conformer state and if this dimerization would affect or modulate the assembly kinetics of Shieldin and Polζ.

**Fig. 3:**
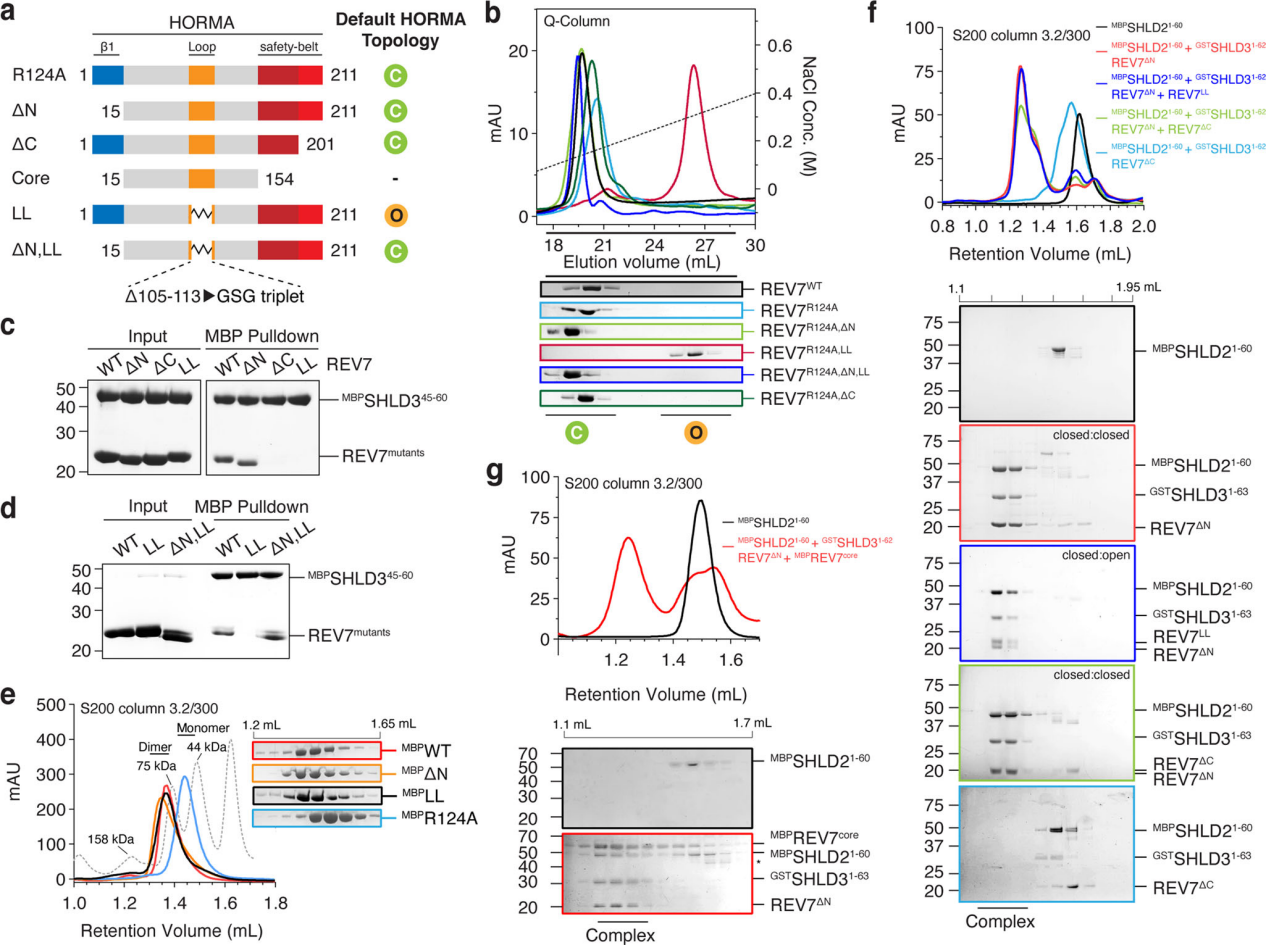
Shieldin assembly requires only one ‘closed’ conformer in REV7 dimer. **a** Schematic drawing of REV7 constructs used in this study to trap REV7 in different topologies. Different colors correspond to different regions of REV7 which are as follows; blue corresponds to residue 1–14, orange (105–113), brick red (155–201) and red (202–211). **b** Anion-exchange chromatography separates monomeric ‘closed’ REV7 from ‘open’ REV7. REV7, REV7^ΔN^, and REV7^ΔC^ elute in the ‘closed’ conformation. REV7^LL^ elutes in the ‘open’ REV7 conformation, which can be restored to the ‘closed’ conformation by the additional removal of the N-terminal 14 residues of REV7 (REV7^ΔN,LL^). The R124A dimerization mutant eliminate potential contributions of dimerization. Eluted fractions were collected and analysed by SDS-PAGE. **c** MBP-SHLD3^45–65^ pulldown showing safety-belt interaction between SHLD3^45–65^ with ‘closed’ REV7 mutants. ‘Open’ REV7 (REV7^LL^) and seat-belt (REV7^ΔC^) mutants fail to bind SHLD3^45–60^. **d** MBP-SHLD3^45–65^ pulldown showing that REV7^LL^ cannot bind SHLD3, however the binding can be restored by the additional removal of the N-terminal 14 residues of REV7 (REV7^ΔN,LL^). **e** REV7 ‘conformer’ mutants (MBP-tagged) elute as dimers from size-exclusion column. The R124A mutant prevents dimerization and serves as monomer control. The consecutive 50 µL fractions eluting from 1.2 and 1.65 mL are shown. **f** Size-exclusion chromatography profiles show interaction between equimolar ratios of MBP-SHLD2^1–60^, REV7^ΔN/ΔC^, GST-SHLD3^1–62^, and REV7^ΔN/ΔC/LL^. Samples were analysed by SDS-PAGE. **g** Size-exclusion chromatography profiles show interaction between MBP-SHLD2^1–60^, REV7^ΔN^, GST-SHLD3^1–62^ and MBP-REV7^core^. MBP-SHLD2^1–60^ and MBP-REV7^core^ were loaded in excess. Samples were analysed by SDS-PAGE.

After reconstituting the Shieldin complex, we used SEC coupled to static angle light scattering equipment (SEC-SLS) to confirm the incorporation of two REV7 molecules. We measured a mass of 90 kDa, which agrees with a theoretical mass of 88 kDa of a 2:1:1 stoichiometric REV7-SHLD3-SHLD2^1–95^ complex (Fig. S2a). The conversion between conformers is defined by substantial unfolding and repositioning of structurally mobile elements at the N- and C-terminus to the static core fold of the HORMA domain. Following a path previously explored with MAD2, we deleted the N- or C-terminal structurally mobile elements of REV7 with the aim to affect the default conformer state (Fig. S2b)^22^. We used a surface-charge based anion-exchange assay to identify and purify separate conformers (Figs. 3a, S2b). The point mutant R124A was used to eliminate any differential elution due to dimerization without affecting the conformer state (Fig. 3b). We observed that all tested REV7 constructs were mono-disperse as they eluted in either of two distinct elution volumes (Fig. 3b). In contrast to previous work, we did not observe conformer mixtures or changes in conformer identity after incubations over prolonged times at different temperatures or buffer conditions in the absence of client proteins^6,16,23^. We therefore concluded that all REV7 variations we created and purified are stable conformers in the unbound state.

REV7^WT^, REV7^R124A^ and REV7^R124A,ΔN^ eluted in the first peak, which was previously been recognized as the SHLD3 binding-competent ‘closed’ state, could indeed interact with a peptide containing the SHLD3^RBM^ (SHLD3^1.62^) (Fig. 3b, c)^6^. REV7^R124A,ΔC^ also eluted in the first peak, suggesting it might be conformationally similar to the ‘closed’ conformer, but the lack of the seatbelt prevented it to interact with SHLD3^1–62^ (Fig. 3c). This is in contrast to MAD2, where deleting a similar C-terminal portion would stabilize MAD2 in the ‘open’ state^21,24,25^. In order to create a REV7 construct that would default to an ‘open’ state, we created a REV7 ‘loopless’ (REV7^LL^) mutant. This mutant was inspired by the elegant MAD2^LL^ mutant, where the shortening of an internal loop traps MAD2 in a stable ‘open’ state^16^. Indeed, REV7^R124A,LL^ was the only REV7 variant that elutes in the distinctly different second peak and cannot interact with SHLD3^1.62^ (Fig. 3c). This second peak is reminiscent of the previously identified conformer that is unable to readily interact with SHLD3^6^. The additional removal of the N-terminal mobile structural element (REV7^R124A,LL,ΔN^) changes its elution back to the first peak, showing that the ability to adopt the ‘closed’ state was restored (Fig. 3c). Indeed, this construct was again able to bind to SHLD3 (Fig. 3d). Overall, this confirms that REV7 can switch between conformers, but defaults to a ‘closed’ conformer. REV7 requires both a functional seatbelt and the ability for this seatbelt to adopt the closed conformation to interact with the RBM on SHLD3^6,14^.

Having established that the first REV7 molecule has to be in the ‘closed state’, we wondered if the incorporation of the second REV7 in Shieldin is also conformer-sensitive. We therefore first tested whether REV7 dimerization is affected by the conformer mutants. Since REV7^WT^ weakly homodimerizes with a K_D_ of ~2 µM, we injected our conformer mutants on a SEC column using a concentration of 20 µM^12^. We used REV7^R124A^ as a monomer control. This showed that all REV7 conformer variants are able to dimerize, which contrasts the conformer-sensitive dimerization observed for MAD2^16,26^ (Figs. 3e, S2c). Next, we used the mutants to test their ability to assemble the Shieldin complex. We mixed purified individual Shieldin components in stoichiometric amounts and used MBP-tagged SHLD2^1–60^ to circumvent stability issues with the full-length SHLD2, and monitored assembly using analytical SEC. As expected, when using mutants that can only be in the ’open’ conformer (REV7^LL^) or that lack the seatbelt (REV7^ΔC^), we could not observe assembly of the Shieldin complex, confirming that at least one REV7 molecule needs a functional seatbelt in the closed conformation (Fig. 3f). In contrast, complex formation was unperturbed when using REV7^ΔN^, REV7^ΔN^-REV7^ΔC^ (both of which could form ‘closed’:’closed’ dimers) or REV7^ΔN^-REV7^LL^ (which putatively combines ‘closed’ and’open’ conformers). This suggests that there is no discrimination between conformer states for the second REV7 molecule, implying that the mobile elements of the second REV7 are not essential (Fig. S2b). To test this, we created a REV7^core^ construct that lacks all mobile elements and therefore cannot adopt the ‘closed’ nor the ‘open’ conformer (Figs. 3a, S2b). As expected, this REV7^core^ construct failed to interact with SHLD3^1–62^ (Fig. S2d). In contrast, when incubated with MBP-SHLD2^1–60^ and a preformed REV7-SHLD3, the REV7^core^ can be incorporated in Shieldin as judged by the shift of the SHLD2-elution peak and co-migration with the other components (Fig. 3g). Taken together, we show that the Shieldin complex can contain a conformationally asymmetric REV7 dimer, that requires one ‘closed’ REV7 bound to SHLD3 and a second REV7 molecule that can adopt any conformation to mediate the interaction with SHLD2 to the static core of REV7.

### Only REV7-SHLD3^RBM^ binding is rate-limiting for Shieldin assembly

**Fig. 4:**
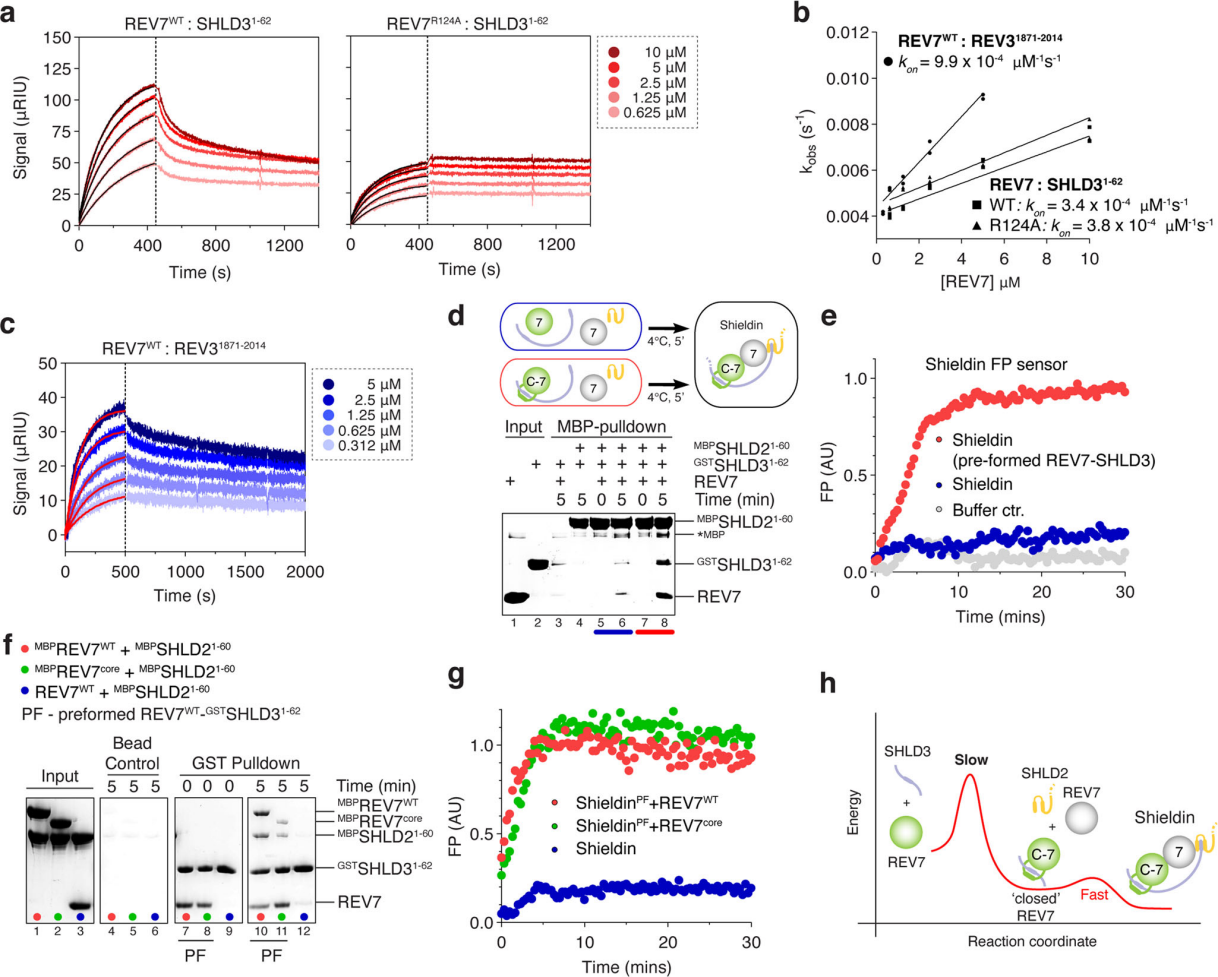
REV7-SHLD3RBM binding is rate limiting for Shieldin assembly. **a** SPR sensorgrams obtained after injection of REV7^WT^ or REV7^R124A^ over immobilized SHLD3^1–62^ on a HC30M chip. Dotted line represents the start of the dissociation phase. **b** Association kinetics of REV7^WT^ and REV7^R124A^ with SHLD3^1–62^ or REV7^WT^ with REV3^1871–2014^. Calculated from sensorgram in (**a**). Due to absence of any appreciable k_off_ (see text), k_on_ was determined by plotting k_obs_ against the concentration of REV7, and determined using linear regression using Graphpad prism. k_obs_ was calculated by fitting (black curves) one-phase association equation assuming 1:1 binding in the binding phase (0–500 s). Individual values plotted and mean values presented as straight line, *n* = 2 independent experiments. **c** SPR sensorgram obtained after injection of REV7^WT^ over immobilized REV3^1871–2014^ on a HC30M chip. Dotted line represents the start of the dissociation phase. **d** Pre-formed REV7-SHLD3 interacts faster with REV7-SHLD2. MBP pulldown showing complex formation at 4 degrees Celsius between MBP-SHLD2^1–60^, REV7, GST-SHLD3^1–62^ or pre-formed GST-SHLD3^1–62^-REV7. **e** FP sensor monitors real time Shieldin complex assembly in vitro. Time-dependent changes in fluorescence polarization (FP) were monitored at room temperature directly after mixing 100 nM Alexa488-labeled SHLD2 with 100 nM SHLD3^1–62^ and 200 nM REV7^WT^. Red curve represents association of preformed REV7-SHLD3 to REV7-SHLD2 whereas blue curve represents association after mixing individual Shieldin components. **f** Incorporation of a second REV7 molecule is fast and conformer independent. GST pulldown showing complex formation between preformed GST-SHLD3^1–62^-REV7, MBP-SHLD2^1–60^, and MBP-REV7^WT^ and MBP-REV7^core^. **g** Incorporation of a second REV7 molecule is fast and conformer independent. Real time assembly of the Shieldin complex was monitored using fluorescence polarization (FP) similar to **e**). Preformed REV7-SHLD3 at 100 nM was incubated with 100 nM SHLD2. Similar rates were observed with addition of either 100 nM REV7^WT^(red) or REV7^core^ (green). **h** Schematic of the proposed Shieldin assembly model. Shieldin assembly requires the slow incorporation of the SHLD3 RBM in a ‘closed’ REV7, followed by faster incorporation of SHLD2 through REV7 dimerization and subsequent recruitment of SHLD2.

The emerging paradigm for HORMA domain proteins is that they default to an inactive ‘open’ state, which is then poised to convert to a ‘closed’ partner-bound active state. This slow, but spontaneous, conversion is a rate-limiting step in the assembly of the respective effector complex. The interaction of a client protein to a pre-closed conformer can also be slow, either because the HORMA domain first needs to (partly) open, or due to slow threading of a sufficiently small enough peptide through the already closed seat-belt^27^. As described in Fig. 3b, the purified REV7 is already in the ‘closed’ state, so potentially no conversion would be necessary. We therefore wondered how the interaction kinetics of a pre-closed apo-REV7 to its client proteins compared to known HORMA domain interaction kinetics. To quantify the binding rates, we used surface plasmon resonance (SPR) with immobilized SHLD3^1–62^ or REV3^1871–2014^ (Fig. 4a–c) and injected either wild-type or dimerization incompetent REV7 (REV7^R124A^). Since complex assembly kinetics are dependent on the concentration of the individual components, we opted to use higher concentrations of REV7 than necessary based on the dissociation constant (Kd = 16 nM^2,14,15^) in order to accelerate the expected slow reaction and to prevent practical measurement issues. We observed the specific binding of REV7^WT^ to SHLD3^1–62^ with a half-binding time (t_1/2_) of 119.6 s at 5 µM REV7, indicative of complex formation that would require many hours to complete at concentrations close to the Kd. This is reminiscent of the slow assembly reported before using qualitative pulldown experiments^7^. When washing with buffer to measure the dissociation of REV7, only a fraction of the signal is lost, suggesting only a partial disassembly of the complex (Fig. 4a, left). Given the high protein concentrations, we anticipated that REV7 might engage as a dimer, after which only one molecule is stably associated with SHLD3. Indeed, when using a dimerization deficient REV7^R124A^, we observed no discernible dissociation in the second phase (Fig. 4a, right). We used the plateau values at the end of the dissociation phase to estimate the binding constant of the REV7-SHLD3^1–62^ interaction to be ~50 nM, in agreement with previous studies^2,14,15^ (Fig. S2e). A stringent washing and regeneration protocol allowed for repeated experiments with increasing concentrations of REV7, which we used to calculate the association constant k_on_ (Fig. 4b). We observed no differences between the wild-type and dimerization mutant of REV7^R124A^, suggesting that dimerization itself does not influence the interaction kinetics of REV7-SHLD3^1.62^. This is reminiscent of the MAD2-mediated assembly of MCC where a dimerization mutant does not affect basal assembly^27^. The binding constants determined for REV7-SHLD3^1–62^ were similar to the interaction kinetics of REV7 to REV3, suggesting that the interaction is not assisted by the client protein interaction motif (Figs. 4b, c, S2f)^12,28^.

Next, we wondered if the incorporation of the second molecule of REV7 used similar interaction kinetics. This second REV7 interaction to SHLD3 is mediated via SHLD2. Indeed, SHLD2 is not sufficient to interact strongly to SHLD3 by itself (Fig. 4d, lane 4), so any interaction to SHLD3 is dependent on the incorporation of the second REV7 molecule. When allowing only a five-minute incubation, SHLD2 can indeed merely incorporate sub-stoichiometric amounts of REV7-SHLD3, due to the slow and incomplete REV7-SHLD3^1–62^ complex formation (Fig. 4d, lane 6). To selectively monitor the second REV7 interaction, we pre-form a stoichiometric REV7-SHLD3^1–62^ complex in the absence of SHLD2, which selectively incorporates only the first REV7 molecule. When mixing the pre-formed complex with additional REV7 and SHLD2, we observed strongly increased amounts of SHLD3 and REV7 in a SHLD2 pulldown after a five-minute incubation, indicating that incorporating the second REV7 is much faster than the first independent of temperature (Fig. 4d, compare lane 6 and 8, and S2g). In order to monitor complex formation in real time, we created a fluorescence polarization sensor (Fig. S2h), where SHLD2 was fluorescently labeled with Alexa488 using the SortaseA transpeptidase enzyme labeling^29^. We observed an increase of fluorescence polarization after an overnight pre-incubation of 100 nM labeled MBP-SHLD2^1–60^ with 0.5 μM of SHLD3^1–62^ and 1 μM REV7, but not when any Shieldin component was omitted (Fig. S2i). This showed that the sensor specifically measures the assembly of the full REV7-SHDL3-SHLD2 complex. We did not observe complex formation under these conditions when using the R124A mutant of REV7, which suggested that the overall complex stability is affected due to the weakened dimerization. We could revert the effect of the mutant by using an increased concentration of REV7 (15 μM instead of 1 μM; Fig. S2j). Next, we monitored complex formation in real time at room temperature by measuring fluorescence polarization after mixing 100 nM Alexa488-labeled SHLD2 with 100 nM SHLD3^1–62^ and 200 nM REV7^WT^ at the start of the reaction (Figs. 4e, S2k). Complex formation required about 6 hours to complete, but this could be accelerated to roughly 10 minutes if the REV7-SHLD3 interaction was already pre-formed at the start of the reaction. Overall, these experiments show that the REV7-SHLD3^RBM^ interaction represent a kinetic bottleneck in Shieldin assembly, while also suggesting that the incorporation of the second REV7 molecule might not be rate-limiting.

Next, we performed experiments to specifically monitor interaction kinetics involving the second REV7 molecule. First, we performed pull down experiments where we pre-form the REV7-SHLD3^1–62^ complex and monitor incorporation of an additional REV7^WT^ or REV7^core^. This showed that the second molecule of REV7 can be incorporated within only 5 minutes, while as a comparison the REV7-SHLD3^1–62^ could not form within this time frame (Fig. 4f). We again used the fluorescence polarization sensor to monitor complex formation, and observed no difference in the ability between REV7^WT^ and REV7^core^ to occupy the position of the second REV7 molecule within Shieldin (Fig. 4g). Overall, this shows that in contrast to the first REV7 interaction, no conformer conversion and no mobile structural elements are necessary for the second REV7 and that the incorporation of the second the REV7 molecule is much faster than the first REV7 interaction with SHLD3^RBM^ and therefore does not affect the assembly kinetics of Shieldin.

## Discussion

Shieldin is an important component of the DNA double-strand break repair machinery, that modulates DNA resection and thereby induces repair via NHEJ. In this role, Shieldin affects the development and treatment of human disease, such as Fanconi Anemia and cancer, in which it determines the sensitivity of BRCA1-deficient cancers to treatment with PARP inhibitors^30^. Given these critical roles, understanding the requirements for proper Shieldin activity is important.

Here, we have shown that a DNA binding domain at the C-terminus of SHLD3 binds a large variety of combinations of both single- and double-strand DNA, which will likely aid the network of DNA and chromatin binding proteins to guide Shieldin recruitment to double-strand breaks. The promiscuous nature of the binding of DNA would provide flexibility for the Shieldin complex to bind a large variety of DNA substrates, including both extensively- and poorly-resected DNA ends. The Shieldin component SHLD2 brings three putative tandem oligonucleotide/oligosaccharide-binding (OB) folds that specifically bind to single-strand DNA, which likely aid to direct the Shieldin complex to the proper DSB site where it can inhibit resection mediated by EXO1 and DNA2/BLM nucleases.

Shieldin is believed to be a dynamic complex that might be hierarchically recruited to sites of DSBs^2,6–8^. Central to the dynamic nature of the Shieldin complex, is the rare ability of REV7 to adopt multiple distinct native states. In surprising contrast to previous work^6^, apo-REV7 in our purifications is exclusively in the ‘closed’ state and we have not been able to induce spontaneous conformational switching to another state in the wild-type protein. It is currently unclear how many conformer states can be adopted by REV7. Structural studies have shown the existence of two states: the canonical ‘closed’ state and a structurally related ‘unbuckled’ version that was named ‘open’^13,28,31^. This latter REV7 conformer can bind SHLD3 and does not structurally resemble the auto-inhibited ‘open’ MAD2, which is the only available structure of a canonical ‘open’ HORMA domain^16^. An auto-inhibited conformer of REV7 has been reported^6^. No structural information is currently available for this REV7 conformer, nor have we been able to purify this conformer, however our data suggests that the N-terminus could be involved in adopting a state that does not readily bind SHLD3 (Fig. 3b–d). Future work will be needed to study Shieldin assembly starting from different purified wild-type conformers in order to deconvolute its full assembly mechanism.

The measured association constants between REV7 and SHLD3^RBM^ are comparable to the interaction kinetics of related HORMA domain proteins, like mitotic protein MAD2 (10^−3^ to 10^−5 ^μM^−1^s^−1^), due to which complex formation can take hours to days at the physiological concentration of the proteins^27,32,33^. Fast interaction kinetics have been observed for MAD2 and REV7 using ITC binding experiments^2,21,26,34,35^. These fast interaction kinetics are likely non-physiological due to the fact that MAD2 and REV7 were present in large excess and high concentrations, which allows for a fraction of the mixture of conformer states to interact quickly to the relatively small amounts of injected interacting peptides.

REV7 captures SHLD3^RBM^ in a seat-belt conformation, which is similar to how MAD2 captures the CDC20 closure motif. The slow interaction between MAD2 and CDC20 is due to the substantial structural remodeling needed for MAD2 to wrap its C-terminal tail around the interacting peptide motif. This reversible structural remodeling is the rate-limiting step that triggers the assembly of the stable but temporary signaling complex (the Mitotic Checkpoint Complex). This suggests that rate-limiting conformational rearrangements are required for Shieldin assembly, also when starting with an already ‘closed’ REV7^27^. External factors like MAD1, BUB1, MPS1 and TRIP13 can accelerate the structural conversion of MAD2, both at the assembly and the disassembly level, allowing dynamic control of signaling^10,27,36–38^. We hypothesize that the same concept is conserved for Shieldin, where the REV7 conformer conversion would be regulated to allow for complex self-assembly or disassembly. Since the c-NHEJ pathway is completed just under 30 min in cells, this argues for the presence of as-of-yet unidentified accelerating factors or modifications of Shieldin assembly^39^.

The transient and asymmetric dimerization between an ‘open’ and ‘closed’ MAD2 is an essential step in the mechanism to accelerate conformer conversion^10,21^. Similar to MAD2, we have not observed changes in Shieldin assembly induced by the dimerization of REV7. Therefore, although dimerization is essential, it is not sufficient to accelerate conversion. In contrast to the transient dimerization of MAD2, the Shieldin complex contains a stable REV7 dimer^7,15,31^. Since a second REV7 could potentially create an additional rate-limiting step in Shieldin assembly, we aim to understand the conformer identity and interaction mechanism of the second REV7. We show that REV7 can form dimers independent of conformer states. Additionally, all these dimers could be incorporated into Shieldin, as long as one REV7 molecule could adopt the ‘closed’ seat-belt interaction. This ‘closed’ REV7 is flanked by a second REV7 in a conformer insensitive manner: it does not require any of the REV7 mobile structural elements, resulting in an unrestricted and fast incorporation in the Shieldin complex. Any external factors that would control the assembly of Shieldin, are likely to modulate the incorporation of the first REV7, and not the second.

AAA^+^-ATPase TRIP13 activity is needed for proper Shieldin function in cells^6,7^. The interaction of TRIP13 to Shieldin, requires a dimer of REV7^7,31^. TRIP13 is a conserved and generic HORMA remodeling factor that can open ‘closed’ HORMA domain proteins in an ATP-dependent manner^38,40^. For example, the opening of closed MAD2 induces the disassembly of the MCC and ‘primes’ the HORMA domains for renewed capture of client proteins by adopting the ‘open’ state. We propose that only the remodeling of the REV7 bound to SHLD3^RBM^ would be necessary to induce Shieldin disassembly, as the interaction of the second REV7 is conformer-independent. The role of TRIP13 co-factor p31 in this mechanism is unclear, as it is not necessary for the interaction of Shieldin to TRIP13 in contrast to MAD2^7^. REV7 and p31 are both HORMA domain proteins, that are reported to weakly interact^12^. Overall, this suggests an exchange mechanism, where p31 could replace the second REV7 in the first step of disassembling Shieldin, after which it would direct TRIP13 to open the remaining ‘closed’ REV7. Alternatively, but not mutually exclusively, TRIP13 could function by converting ‘closed’ apo-REV7 to adopt the ‘open’ conformer, which might require p31 as apo-REV7 dimerization is relatively weak.

Overall, this study provides insights into Shieldin recruitment and assembly. SHLD3 could serve as the platform to recruit the first molecule of REV7. The RBM of SHLD3 is captured by REV7 using a seat-belt interaction mechanism, which represents a rate-limiting step in the assembly of Shieldin. After overcoming this obligatory step, Shieldin self-assembly is fast using a cooperative mechanism within REV7-SHLD2-3 (Fig. 4h). Future work will be required to find factors that modulate the conversion of REV7 conformers in a timely fashion and to specifically regulate the assembly of its many client effector complexes in different pathways.

## Methods

### Expression of recombinant proteins and purification

All recombinant proteins used in this study were of human origin. REV7 mutants, REV3^1871–2014^, SHLD2 N-terminal, and SHLD3 N- and C-terminal constructs were expressed with an N-terminal hexahistidine-MBP or GST fusion-tag from pLIB at 16 °C in *E.coli* LOBSTR strain^41^ for 16 hr after induction with 0.1 mM IPTG. Cells were lysed by sonication in buffer A containing 25 mM HEPES-NaOH (pH 7.5), 0.3 M NaCl, 0.5 mM TCEP (VWR lifesciences) and 1 mM PMSF (Roche). After clearing, the lysate was loaded on a Hi-Trap metal chelating column (Cytiva). Bound proteins were eluted with an imidazole gradient. The MBP tag or GST tag was cleaved from protein constructs using PreScission protease overnight and subsequently separated using reverse-affinity purification. Protein containing fractions were pooled, and concentrated in 10 kDa MWCO concentrator (Merck) and loaded onto a Superdex-75 column (GE Healthcare) equilibrated with buffer B containing 10 mM HEPES-NaOH pH 7.5, 0.15 M NaCl and 0.5 mM TCEP for size-exclusion chromatography (SEC). Fractions containing purified REV7, REV3, SHLD2 and SHLD3 constructs were concentrated, flash-frozen and stored at −80 °C until use. REV7 (both R124A and WT)-SHLD3-SHLD2^1–95^ complex was purified from insect cell using biGBac expression system with GST fused to SHLD2^42^. Bacmid produced from DH10EMBacY cells was used to transfect Sf9 cells and produce baculovirus. Baculovirus was amplified through three rounds of amplification and used to infect Hi5 cells. Cells infected with the viruses were cultured for 72 h before harvesting. Purification of protein complexes was carried out using above protocol. Further polishing of the complex prior SEC and after tag cleavage with PreScission protease was carried out by loading onto a cation-exchange (CE) Resource-S column (GE Healthcare) equilibrated in 10 mM HEPES (pH 7.5), 50 mM NaCl and 0.5 mM TCEP. Elution was carried out using 0.05–1 M NaCl gradient over 20 column volumes. Fractions containing purified REV7 (both R124A and WT)-SHLD3-SHLD2^1–95^ were concentrated, flash-frozen and stored at −80 °C until use.

### In vitro binding assays

For MBP-pulldown experiments, 1 μM MBP-SHLD3^45–60^ pre-adsorbed on Amylose beads was incubated for 30 min at 4 °C with 2 μM of REV7, REV7^LL^, REV7^ΔN^, REV7^ΔN,LL^ and REV7^ΔC^ in buffer B. After two washing steps of 0.5 ml each with buffer containing 10 mM HEPES (pH7.5), 0.5 M NaCl, 5% glycerol and 0.5 mM TCEP, complexes immobilized on beads were mixed with SDS gel loading buffer and analyzed by 12% SDS-PAGE. For GST-pulldown experiments, 1 μM GST-SHLD3^1–63^-REV7 pre-adsorbed on glutathione beads was incubated for 5 min at 4 °C with 2 μM of MBP-REV7^core^ or MBP-REV7 and 2 μM MBP-SHLD2^1–60^ in buffer B. After two washing steps of 0.5 ml each with buffer containing 10 mM HEPES (pH 7.5), 0.01% Tween 20, 0.5 M NaCl, 5% glycerol and 0.5 mM TCEP, complexes immobilized on beads were mixed with SDS gel loading buffer and analyzed by 12% SDS-PAGE.

### Static angle light scattering measurement

The SLS measurement was performed by coupling Superdex-200 column with VISCOTEK 305 TDA detector (Malvern). The run was performed in buffer B with prior calibration using BSA. The scattering was measured at 90° (right angle scattering) and 7° (low angle scattering) and data evaluation was carried out using OmniSEC v5.12 software (Malvern).

### Anion-exchange chromatography

Purified REV7 mutants at 500 µg in total were subjected to anion-exchange (AE) chromatography using a 1 ml Hi-Trap Q column (Cytiva). Protein samples were resuspended in low salt buffer B prior loading onto Q column. Bound proteins were eluted using a 20–500 mM NaCl gradient over 20 column volumes. Fractions collected from the run were analyzed by 12% SDS-PAGE.

### Analytical size-exclusion chromatography

SEC runs were performed on Superdex-200 column equilibrated with buffer C (20 mM Na-HEPES pH 7.5, 5% (v/v) glycerol, 100 mM NaCl and 0.5 mM TCEP). Prior run, SHLD3-SHLD2^1–95^-REV7^R124A^ complex and 5,6-FAM-labeled 50 bp ssDNA with the following sequence 5′-AAG GGG AGC GGG GGA GGA TAA TAG GAA GGG GAG CGG GGG AGG ATA ATA GG-3′ was incubated for 30 min on ice in dark. Fractions collected from SEC run were analyzed by 12% SDS-PAGE and scanned at 520 nm on Amersham Imager 680. For the REV7 dimerization experiments, 20 µM of REV7 or MBP-REV7 constructs were loaded on Superdex-200 3.2/300 column equilibrated with buffer B. Fractions collected during SEC were analyzed by 12% SDS-PAGE. For Shieldin assembly experiments, 5 µM of MBP-SHLD2^1–60^, GST-SHLD3^1–62^, and REV7 mutants were incubated on ice for 60 min prior loading on the Superdex-200 3.2/300 column equilibrated with buffer B. Fractions collected during SEC run were analyzed by 12% SDS-PAGE. For Shieldin assembly using REV7^core^ construct, 5 µM of preformed REV7^ΔN^-SHLD3 was incubated with 10 µM of MBP-SHLD2^1–60^ and MBP-REV7^core^ on ice for 30 min prior loading on the Superdex-200 3.2/300 column equilibrated with buffer B. Fractions collected during SEC run were analyzed by 12% SDS-PAGE.

### Limited proteolysis of Shieldin complex

Trypsin (Hampton Research) was used for limited proteolysis at 1:100 (trypsin:protein) molar ratio. Protein complex at 10 µM was mixed with trypsin at 0.1 µM in buffer B. Digested samples were taken out at 0, 5, 15, 30 and 60 min. The reaction was quenched by adding 4x SDS gel loading dye and analysed by 15% SDS-PAGE.

### Fluorescence anisotropy

A 5,6-FAM-labeled ssDNA probe with the following sequence 5′-AGT GCC AGT GCC-3′ was purchased from Integrated DNA Technologies and was dissolved in deionized water. The dsDNA probe was generated by heating to 90 °C and then slowly annealing equimolar amounts of 5′-GGC ACT GGC ACT-3′ with FAM-labeled 5′-AGT GCC AGT GCC-3′. For binding affinity measurements, SHLD3-SHLD2^1–95^-REV7^R124A^/ SHLD3^140–250^ was diluted in half-log steps in buffer C. Nucleic acids at a final concentration of 10 nM and SHLD3-SHLD2^1–95^-REV7^R124A^/SHLD3^140–250^ at a concentration range 0-56 µM were mixed on ice. The reaction was brought to a final volume of 25 µL and incubated in dark for 30 mins at room temperature. 18 µL of the reaction mixture was transferred to a Greiner 384 Flat bottom small volume plate. Fluorescence anisotropy was measured with an excitation wavelength of 470 ± 5 nm, an emission wavelength of 518 ± 5 nm, and a gain of 56. Each experiment was performed in triplicates and data was analysed using Graphpad Prism version 8. Binding curves were fit using a one site - specific binding equation. For anisotropy measurements, proteins at 20 µM were incubated with 10 nM of DNA probes. The reaction was brought to a final volume of 25 µL and incubated in dark for 30 mins at room temperature. The measurements were carried out as stated above. Statistical significance was performed using two-tailed student’s *t* test. Each experiment was performed at least in triplicates and data was analysed using Graphpad Prism version 8.

### Surface plasmon resonance measurements

Kinetic measurements were performed on a 2SPR Dual Channel system (XanTec bioanalytics GmbH). Purified SHLD3^1–62^ was immobilized on SCR HC30M chip using EDC-NHS coupling reaction. REV7^WT/R124A^ in two-fold dilutions was injected and analysed. Similar set-up was used for REV7-REV3 protein system with REV3^1871–2014^ immobilized on SCR HC30M chip. Buffer B supplemented with 0.05% Tween 20 (BIO-RAD) was used as a running buffer throughout SPR measurements. Data analysis was carried out using Trace Drawer software (XanTec bioanalytics GmbH).

### AlexaFluor488 labeling of SHLD2

MBP-SHLD2^1–60^ containing C-terminal LPETGG motif was labeled using sortase A transpeptidase enzyme from *Staphylococcus aureus*. Labeling was carried out by mixing 100 μM MBP-SHLD2^1–60^ with 10 μM Sortase A enzyme and 0.5–1 mM of AlexaFluor488-conjugate peptide (GlyGlyGlyCys-AlexaFluor488). The conjugation of AlexaFluor488 to the peptide was carried out by incubating GGGC peptide (GenScript) with AlexFluor488-maleimide (Thermo Fisher Scientific) in 100 mM HEPES-NaOH pH 7.4 at equimolar ratio at 25 °C for 2 h. The labeling reaction was carried out with overnight incubation in buffer B supplemented with 10 mM CaCl_2_ at 4 °C. Labeled MBP-SHLD2^1–60^ was separated from excess AlexaFLuor488-conjugate peptide by SEC (Superdex-75, Cytiva).

### Fluorescence polarization

Fluorescence polarization was measured using a Synergy Neo2 multi-mode reader (BioTek) in buffer B supplemented with 0.05% Tween 20. The experiments were performed at protein concentration of 100 nM for SHLD2 and SHLD3 and 200 nM for REV7 in 100 μL total volume using Greiner 96 well flat bottom μclear plate. Preformed REV7-SHLD3 was prepared by incubating equimolar amounts of REV7^WT^ and SHLD3^1–62^ overnight at 4 °C. All panels reporting time-dependent changes in polarization signal are single measurements representative of three independent technical replicates of the experiments. For end-point polarization measurements, proteins at 0.5–1 μM were incubated with 100 nM of labeled MBP-SHLD2^1–60^ in buffer B supplemented with 0.05% Tween 20. The reaction was brought to a final volume of 25 μL and incubated overnight in dark at 4 °C. 18 μL of the reaction mixture was transferred to a Greiner 384 Flat bottom small volume plate. Fluorescence polarization was measured with an excitation wavelength of 470 ± 5 nm, an emission wavelength of 518 ± 5 nm, and a gain of 56. Each experiment was performed in triplicates and data was analysed using Graphpad Prism version 8.

### Statistics and reproducibility

Statistical analyses were performed with GraphPad Prism using the two-tailed student’s *t* test. Significance; ns not significant (*p* ≥ 0.05); *p < 0.05; **p < 0.01; ***p < 0.001; ****p < 0.0001. Unless indicated otherwise, all gels are representative of at least two independent experiments, with uncropped gels shown in the Supplementary Fig. S3.

### Reporting summary

Further information on research design is available in the Nature Portfolio Reporting Summary linked to this article.

## Supplementary information

Supplementary Information

Reporting Summary

## Data Availability

All data generated or analysed during this study are included in this published article (and its Supplementary Information files). The uncropped SDS-PAGE gels can be found in Supplementary Fig. S3.

## References

[1] Noordermeer SM (2018). The shieldin complex mediates 53BP1-dependent DNA repair. Nature.

[2] Gupta R (2018). DNA Repair Network Analysis Reveals Shieldin as a Key Regulator of NHEJ and PARP Inhibitor Sensitivity. Cell.

[3] Dev H (2018). Shieldin complex promotes DNA end-joining and counters homologous recombination in BRCA1-null cells. Nat. Cell Biol..

[4] Ghezraoui H (2018). 53BP1 cooperation with the REV7-shieldin complex underpins DNA structure-specific NHEJ. Nature.

[5] Setiaputra, D. & Durocher, D. Shieldin—the protector of DNA ends. *EMBO Rep.* **20**, 10.15252/embr.201847560 (2019). 10.15252/embr.201847560 PMC6501030 30948458

[6] Clairmont CS (2020). TRIP13 regulates DNA repair pathway choice through REV7 conformational change. Nat. Cell Biol..

[7] de Krijger I (2021). MAD2L2 dimerization and TRIP13 control shieldin activity in DNA repair. Nat. Commun..

[8] Heyza, J. R., Mikhova, M., Bahl, A., Broadbent, D. & Schmidt, J. C. Systematic analysis of the molecular and biophysical properties of key DNA damage response factors. *bioRxiv*10.1101/2022.06.09.495359 (2022). 10.7554/eLife.87086 PMC10319438 37341699

[9] Gu, Y., Desai, A. & Corbett, K. D. Evolutionary Dynamics and Molecular Mechanisms of HORMA Domain Protein Signaling. *Annu. Rev. Biochem.*10.1146/annurev-biochem-090920-103246 (2022). 10.1146/annurev-biochem-090920-103246 35041460

[10] Faesen AC (2017). Basis of catalytic assembly of the mitotic checkpoint complex. Nature.

[11] Musacchio A (2015). The Molecular Biology of Spindle Assembly Checkpoint Signaling Dynamics. Curr. Biol..

[12] Rizzo AA (2018). Rev7 dimerization is important for assembly and function of the Rev1/Polzeta translesion synthesis complex. Proc. Natl Acad. Sci. U.S.A..

[13] Malik R (2020). Structure and mechanism of B-family DNA polymerase zeta specialized for translesion DNA synthesis. Nat. Struct. Mol. Bio.l.

[14] Dai Y (2020). Structural basis for shieldin complex subunit 3-mediated recruitment of the checkpoint protein REV7 during DNA double-strand break repair. J. Biol. Chem..

[15] Liang L (2020). Molecular basis for assembly of the shieldin complex and its implications for NHEJ. Nat. Commun..

[16] Mapelli M, Massimiliano L, Santaguida S, Musacchio A (2007). The Mad2 conformational dimer: structure and implications for the spindle assembly checkpoint. Cell.

[17] Ashkenazy H (2016). ConSurf 2016: an improved methodology to estimate and visualize evolutionary conservation in macromolecules. Nucleic Acids Res..

[18] Erdos G, Pajkos M, Dosztanyi Z (2021). IUPred3: prediction of protein disorder enhanced with unambiguous experimental annotation and visualization of evolutionary conservation. Nucleic Acids Res..

[19] Jumper J (2021). Highly accurate protein structure prediction with AlphaFold. Nature.

[20] Mirdita M (2022). ColabFold: making protein folding accessible to all. Nat. Methods.

[21] De Antoni A (2005). The Mad1/Mad2 complex as a template for Mad2 activation in the spindle assembly checkpoint. Curr. Biol..

[22] Mapelli M, Musacchio A (2007). MAD contortions: conformational dimerization boosts spindle checkpoint signaling. Curr. Opin. Struct. Biol..

[23] Luo X (2004). The Mad2 spindle checkpoint protein has two distinct natively folded states. Nat. Struct. Mol. Biol..

[24] Fang G, Yu H, Kirschner MW (1998). The checkpoint protein MAD2 and the mitotic regulator CDC20 form a ternary complex with the anaphase-promoting complex to control anaphase initiation. Genes Dev..

[25] Luo X (2000). Structure of the Mad2 spindle assembly checkpoint protein and its interaction with Cdc20. Nat. Struct. Biol..

[26] Yang M (2008). Insights into mad2 regulation in the spindle checkpoint revealed by the crystal structure of the symmetric mad2 dimer. PLoS Biol..

[27] Piano V (2021). CDC20 assists its catalytic incorporation in the mitotic checkpoint complex. Science.

[28] Hara K (2010). Crystal structure of human REV7 in complex with a human REV3 fragment and structural implication of the interaction between DNA polymerase zeta and REV1. J. Biol. Chem..

[29] Popp MW, Ploegh HL (2011). Making and breaking peptide bonds: protein engineering using sortase. Angew Chem. Int. Ed Engl.

[30] Noordermeer SM, van Attikum H (2019). PARP Inhibitor Resistance: A Tug-of-War in BRCA-Mutated Cells. Trends Cell Biol..

[31] Xie W (2021). Molecular mechanisms of assembly and TRIP13-mediated remodeling of the human Shieldin complex. Proc. Natl Acad. Sci. U.S.A..

[32] Simonetta M (2009). The influence of catalysis on mad2 activation dynamics. PLoS Biol..

[33] Vink M (2006). In vitro FRAP identifies the minimal requirements for Mad2 kinetochore dynamics. Curr. Biol..

[34] Sironi L (2002). Crystal structure of the tetrameric Mad1-Mad2 core complex: implications of a ‘safety belt’ binding mechanism for the spindle checkpoint. EMBO J..

[35] Sironi L (2001). Mad2 binding to Mad1 and Cdc20, rather than oligomerization, is required for the spindle checkpoint. EMBO J..

[36] Kim DH (2018). TRIP13 and APC15 drive mitotic exit by turnover of interphase- and unattached kinetochore-produced MCC. Nat. Commun..

[37] Lara-Gonzalez P, Kim T, Oegema K, Corbett K, Desai A (2021). A tripartite mechanism catalyzes Mad2-Cdc20 assembly at unattached kinetochores. Science.

[38] Ye Q (2015). TRIP13 is a protein-remodeling AAA+ ATPase that catalyzes MAD2 conformation switching. Elife.

[39] Mao Z, Bozzella M, Seluanov A, Gorbunova V (2008). Comparison of nonhomologous end joining and homologous recombination in human cells. DNA Repair.

[40] Alfieri C, Chang L, Barford D (2018). Mechanism for remodelling of the cell cycle checkpoint protein MAD2 by the ATPase TRIP13. Nature.

[41] Andersen KR, Leksa NC, Schwartz TU (2013). Optimized E. coli expression strain LOBSTR eliminates common contaminants from His-tag purification. Proteins.

[42] Weissmann F (2016). biGBac enables rapid gene assembly for the expression of large multisubunit protein complexes. Proc. Natl Acad. Sci. U.S.A..

[43] Waterhouse AM, Procter JB, Martin DM, Clamp M, Barton GJ (2009). Jalview Version 2–a multiple sequence alignment editor and analysis workbench. Bioinformatics.

