# Supplementary material for: Shieldin complex assembly kinetics and DNA binding by SHLD3
Source: Commun Biol. 2023 Apr 8;6:384. doi: 10.1038/s42003-023-04757-7 (PMC10082759; doi:10.1038/s42003-023-04757-7)
Supplement: Supplementary file 1 — Supplementary Information [file 42003_2023_4757_MOESM1_ESM.pdf]

Shieldin complex assembly kinetics and DNA binding by SHLD3

- Supplemental tables and figures -

Vivek Susvirkar and Alex C. Faesen#

Biochemistry of Signal Dynamics, Max-Planck Institute for Multidisciplinary Sciences, Am Fassberg 11, 37077 Göttingen, Germany

#Corresponding Author:

Alex C. Faesen

Biochemistry of Signal Dynamics

Max-Planck Institute for Multidisciplinary Sciences

Am Fassberg 11

37077 Göttingen

Germany

Email: afaesen@mpinat.mpg.de

Phone: +49 551 201-1155

Supplementary Table 1. DNA substrate list for Fluorescence anisotropy experiments

Substrate list	Labelled oligonucleotide	Annealed oligonucleotide
dsTelo	1	2
dsNTelo	3	4
ssTelo	1	
ssNTelo	3	
15ds15ss(Foldback, 3‘-tail)	6	
15ds15ss(3‘-tail)	7	8
15ds15ss(5‘-tail)	7	9
11ds1ss	3	10
10ds2ss	3	11
9ds3ss	3	12

Supplementary Table 2. List of DNA oligos used in this study

Oligo number	Sequence (5’-3’)
1	TTAGGGTTAGGG
2	CCCTAACCCTAA
3	AGTGCCAGTGCC
4	GGCACTGGCACT
5	AAGGGGAGCGGGGGAGGATAATAGGAAGGGGAGCGGGGGAGGATAATAG G
6	TCCCCTTCCTACTTTTTTGTAGGAAGGGGAGCGGGGGAGGATAATAGG
7	GTAGGAAGGGGAGCGGGGGAGGATAATAGG
8	CGCTCCCCTTCCTAC
9	CCTATTATCCTCCCC
10	GCACTGGCACT
11	CACTGGCACT
12	ACTGGCACT

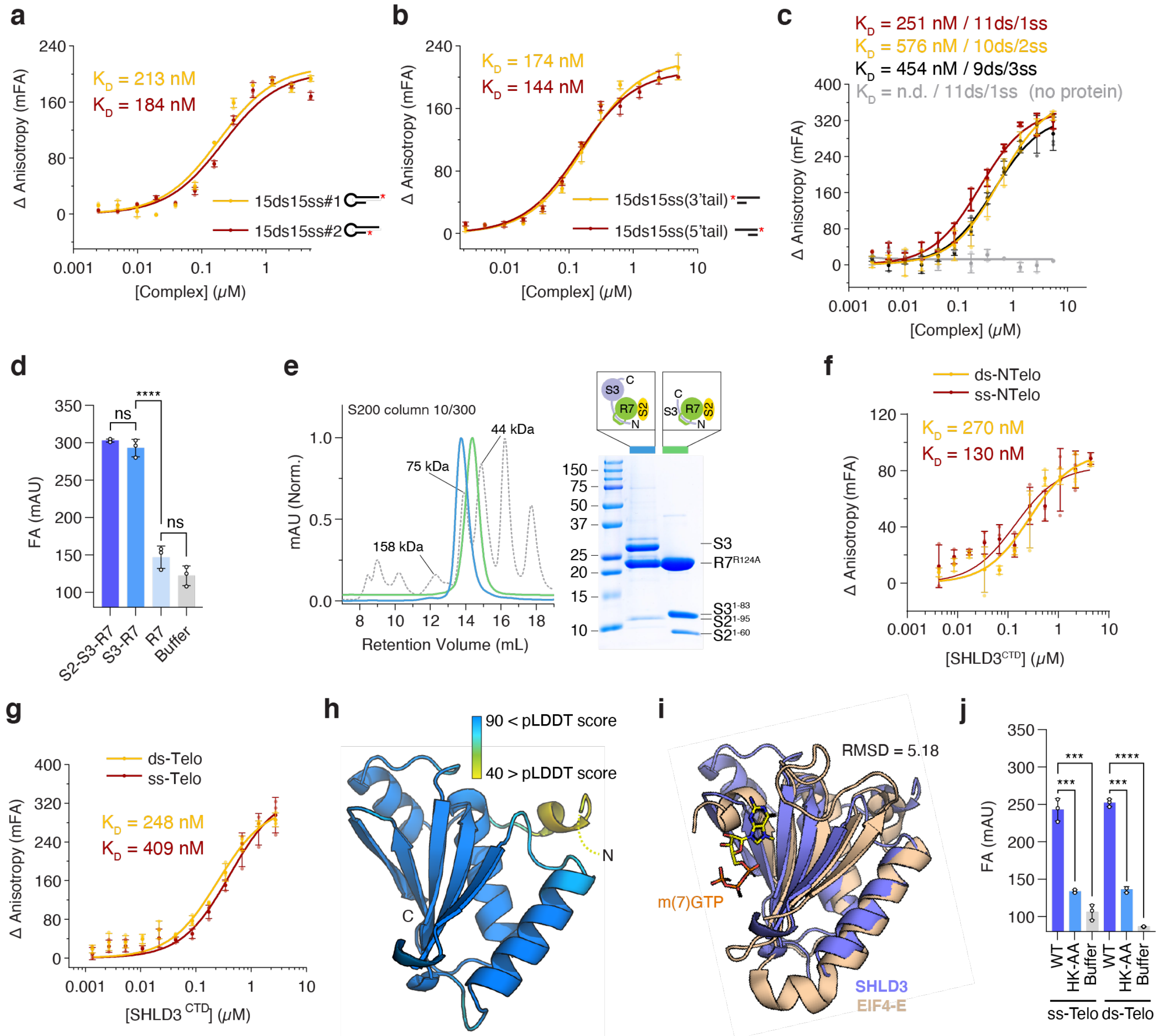

Figure S1. SHLD3 is a DNA binding protein. a-c) SHLD3 shows no preference for DNA substrates. Fluorescence anisotropy titration experiments of SHLD3-SHLD2$^{1-90}$-REV7 complex to determine binding affinities to (a) foldback 15ds15ss overhangs, (b) 15-bp 3'-tailed ssDNA (yellow) or 15-bp 5'-tailed ssDNA (brown), and (c) short 1 to 3-bp ssDNA overhang. The negative control in (c) is the 11ds1ss DNA construct without protein. Asterisks denote conjugated 5,6-FAM dye. Error bars represent s.d. (n = 3 independent experiments). **d)** Fluorescence anisotropy measurements of Shieldin complex, SHLD3-REV7, and REV7 for non-telomeric ssDNA substrates. Error bars represent s.d. (n = 3 independent experiments). Two-tailed Student's test are indicated: ns, not significant, ****p<0.0001. **e)** Purification of stable SHLD3-SHLD2$^{1-95}$-REV7 (blue curve) and SHLD3$^{1-83}$-SHLD2$^{1-60}$-REV7 (green curve) complex by size exclusion chromatography **f,g)** Fluorescence anisotropy titration experiments of SHLD3^{CTD} to determine binding affinities for (f) non-telomeric ss- and dsDNA and (g) telomeric ss- and dsDNA. Error bars represent s.d. (n = 3 independent experiments). **h)** AlphaFold2 predicts a folded SHLD3 C-terminus with a very high confidence (pLDDT > 90). **i)** Structural alignment of SHLD3^{CTD} and human EIF4-E (PDB: 5BXV) shows SHLD3^{CTD} adopts a fold similar to nucleotide binding translation factor. Proteins are coloured as follows SHLD3^{CTD} (marine blue), EIF4-E (beige), and m(7)GTP shown in stick representation. **j)** Fluorescence anisotropy measurements of SHLD3^{CTD} wildtype and HK-AA mutant for telomeric ss- and dsDNA substrates. FAM-labelled ssDNA at 10 nM was incubated with 1 µM SHLD3^{CTD}. Error bars represent s.d. (n = 3 independent experiments). Two-tailed Student's test are indicated: ***p<0.001, ****p<0.0001.

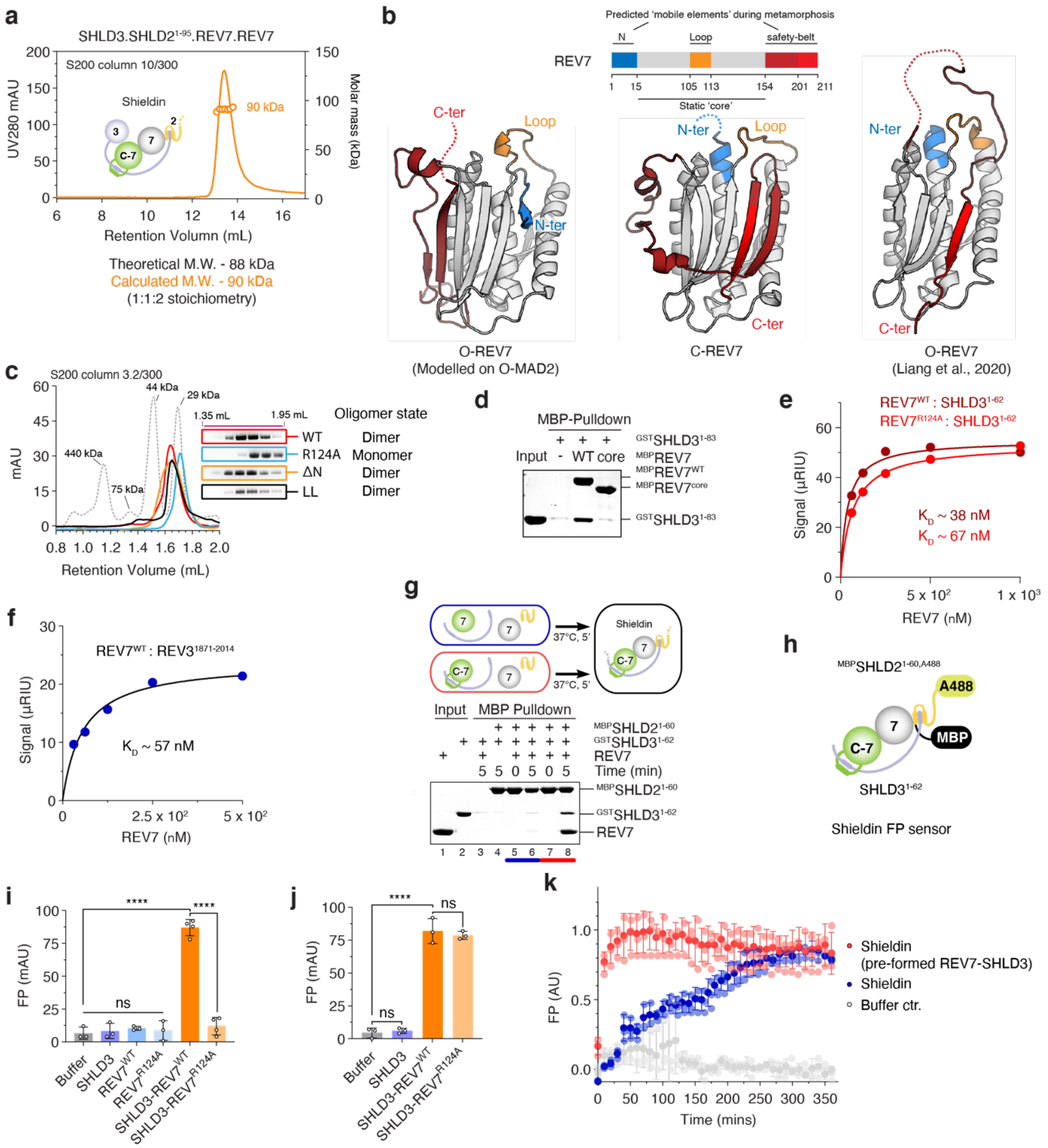

Figure S2. Differential association of the two REV7 molecules within Shieldin. a) Molecular mass determination of the SHLD3-SHLD2$^{1-95}$-REV7 ternary complex using static angle light scattering coupled to size exclusion chromatography. Calculated molar mass of ~ 90 kDa confirms a dimer of REV7 present for each monomer of SHLD3 and SHLD2. **b)** Structural model of a 'open' REV7 based on 'open' MAD2, the crystal structure of 'closed' REV7 and 'open' REV7 as named by Liang et al[1]. Conversion between conformers requires major structural rearrangement of N-terminal region (blue) and C-terminal safety belt region (red). The conversion requires the N-terminal region to thread through the loop region (orange). Inability to thread locks REV7 in the 'open' conformer. The 'open' REV7 structure from Liang et al., shows C-terminus (brick red, residues 201-211) in an 'unbuckled' position with about 30 residues (157 to 190) of the safety-belt region unbound to the core structure. In the modelled

alternative ‘open’ REV7 (left) the C-terminus is positioned across the core β-sheets. **c)** REV7 ‘conformer’ mutants (untagged) elute as dimers from size exclusion column. The R124A mutant prevents dimerization and serves as monomer control. The contents of consecutive 50 μL fractions eluting from 1.35 and 1.95 mL are shown using a SDS PAGE Coomassie-stained gel. **d)** MBP pulldown showing the interaction between GST-SHLD3$^{1-83}$ with MBP-REV7^{WT} and MBP-REV7^{core} mutants. REV7^{core} fails to bind GST-SHLD3$^{1-83}$. **e,f)** Determination of binding affinity of (e) SHLD3$^{1-62}$ to REV7^{WT} or REV7^{R124A} and (f) REV3$^{1871-2014}$ to REV7^{WT}. Plot shows the plateau values at the end of the dissociation phase of the SPR experiments shown in Figure 4a,c. **g)** Pre-formed REV7-SHLD3 interacts faster with REV7-SHLD2. MBP pulldown showing interaction kinetics at 37 degrees Celsius between MBP-SHLD2$^{1-60}$, REV7, GST-SHLD3$^{1-62}$ or preformed GST-SHLD3$^{1-62}$-REV7. **h)** Schematic representation of fluorescence polarization sensor for Shieldin assembly. The C-terminus of MBP-SHLD2$^{1-60}$ is labelled with fluorophore Alexa488 using Sortase coupling[2]. **i,j)** Fluorescence polarization measurement for Shieldin assembly. 0.5 μM of SHLD3$^{1-62}$ and (i) 1 μM or (j) 15 μM of REV7 were incubated overnight with 100nM labelled MBP-SHLD2$^{1-60}$. The sensor shows polarization only in presence of both SHLD3 and REV7^{WT} but not REV7^{R124A}. Error bars represent s.d. (n = 3 independent experiments). Two-tailed Student’s test are indicated: ****p<0.0001. **k)** FP sensor shows that assembly of the Shieldin *in-vitro* at room temperature takes about 6 hours to complete (blue), but can be dramatically accelerated if the REV7-SHLD3 interaction is pre-formed (red). FP measurements were carried out by incubating 100 nM labelled SHLD2 with 100 nM SHLD3$^{1-62}$ and 200 nM REV7^{WT}. Error bars represent s.d. (n = 3 independent experiments).

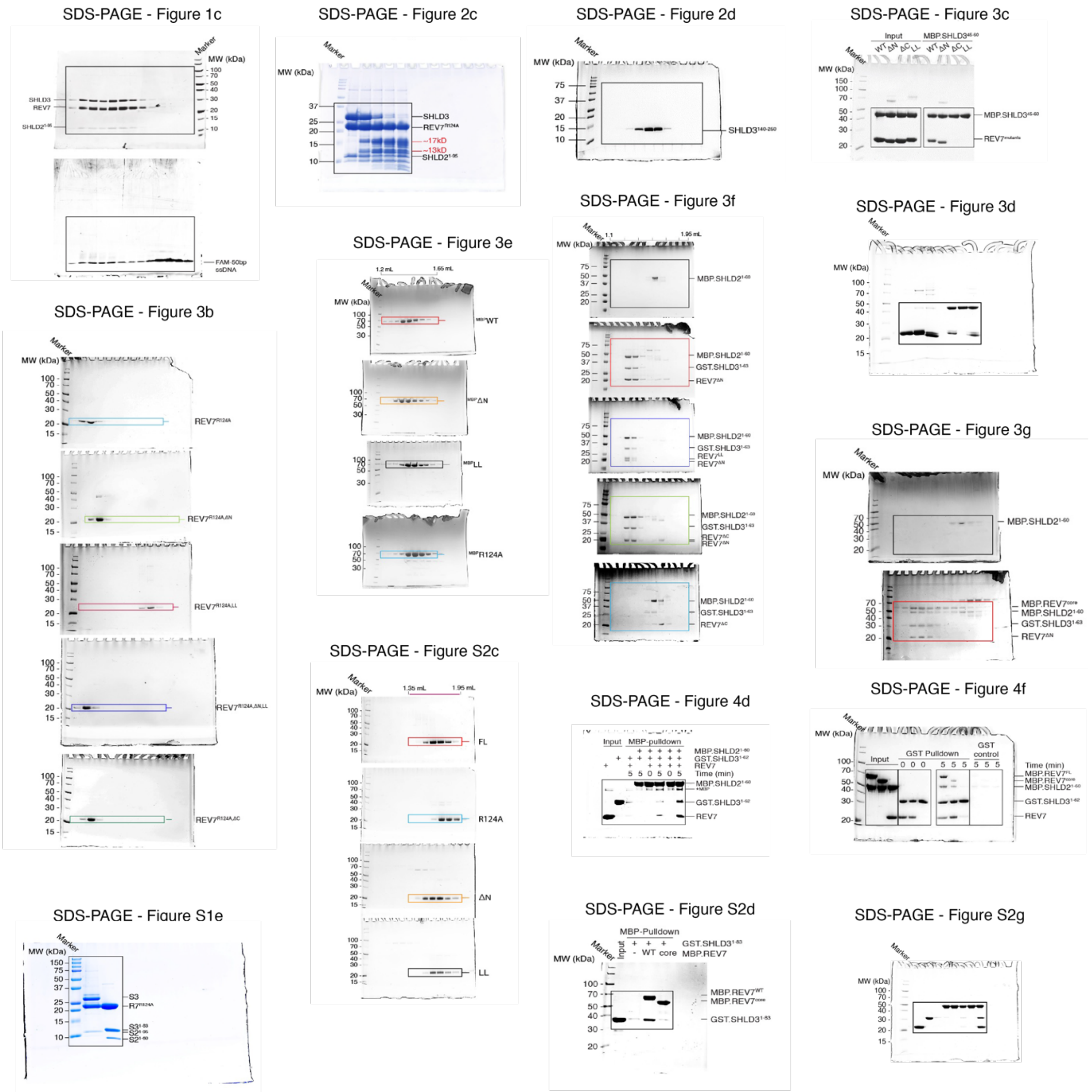

Figure S3. Uncropped SDS-PAGE gels.

References

1 Liang, L. *et al.* Molecular basis for assembly of the shieldin complex and its implications for NHEJ. *Nat Commun* **11**, 1972, doi:10.1038/s41467-020-15879-5 (2020).

2 Popp, M. W. & Ploegh, H. L. Making and breaking peptide bonds: protein engineering using sortase. *Angew Chem Int Ed Engl* **50**, 5024-5032, doi:10.1002/anie.201008267 (2011).
